# Retaining Race in Chronic Kidney Disease Diagnosis and Treatment

**DOI:** 10.7759/cureus.45054

**Published:** 2023-09-11

**Authors:** Paul Williams

**Affiliations:** 1 Life Sciences, Lawrence Berkeley National Laboratory, Berkeley, USA

**Keywords:** egfr creatinine, end stage renal disease (esrd), race-based differences, estimated glomerular filtration rate (egfr), chronic kidney disease (ckd)

## Abstract

The best overall measure of kidney function is glomerular filtration rate (GFR) as commonly estimated from serum creatinine concentrations (eGFR_cr_) using formulas that correct for the higher average creatinine concentrations in Blacks. After two decades of use, these formulas have come under scrutiny for estimating GFR differently in Blacks and non-Blacks. Discussions of whether to include race (Black vs. non-Black) in the calculation of eGFR_cr_ fail to acknowledge that the original race-based eGFR_cr_ provided the same CKD treatment recommendations for Blacks and non-Blacks based on directly (exogenously) measured GFR. Nevertheless, the *National Kidney Foundation* and the *American Society of Nephrology Task Force on Reassessing the Inclusion of Race in Diagnosing Kidney Disease* removed race in CKD treatment guidelines and pushed for the immediate adoption of a race-free eGFR_cr_ formula by physicians and clinical laboratories. This formula is projected to negate CKD in 5.51 million White and other non-Black adults and reclassify CKD to less severe stages in another 4.59 million non-Blacks, in order to expand treatment eligibility to 434,000 Blacks not previously diagnosed and to 584,000 Blacks previously diagnosed with less severe CKD. This review examines: 1) the validity of the arguments for removing the original race correction, and 2) the performance of the proposed replacement formula. Excluding race in the derivation of eGFR_cr_ changed the statistical bias from +3.7 to -3.6 ml/min/1.73m^2^ in Blacks and from +0.5 to +3.9 in non-Blacks, i.e., promoting CKD diagnosis in Blacks at the cost of restricting diagnosis in non-Blacks. By doing so, the revised eGFR_cr_ greatly exaggerates the purported racial disparity in CKD burden. Claims that the revised formulas identify heretofore undiagnosed CKD in Blacks are not supported when studies that used kidney failure replacement therapy and mortality are interpreted as proxies for baseline CKD. Alternatively, a race-stratified eGFR_cr_ (i.e., separate equations for Blacks and non-Blacks) would provide the least biased eGFR_cr_ for both Blacks and non-Blacks and the best medical treatment for all patients.

## Introduction and background

**Figure 1:**
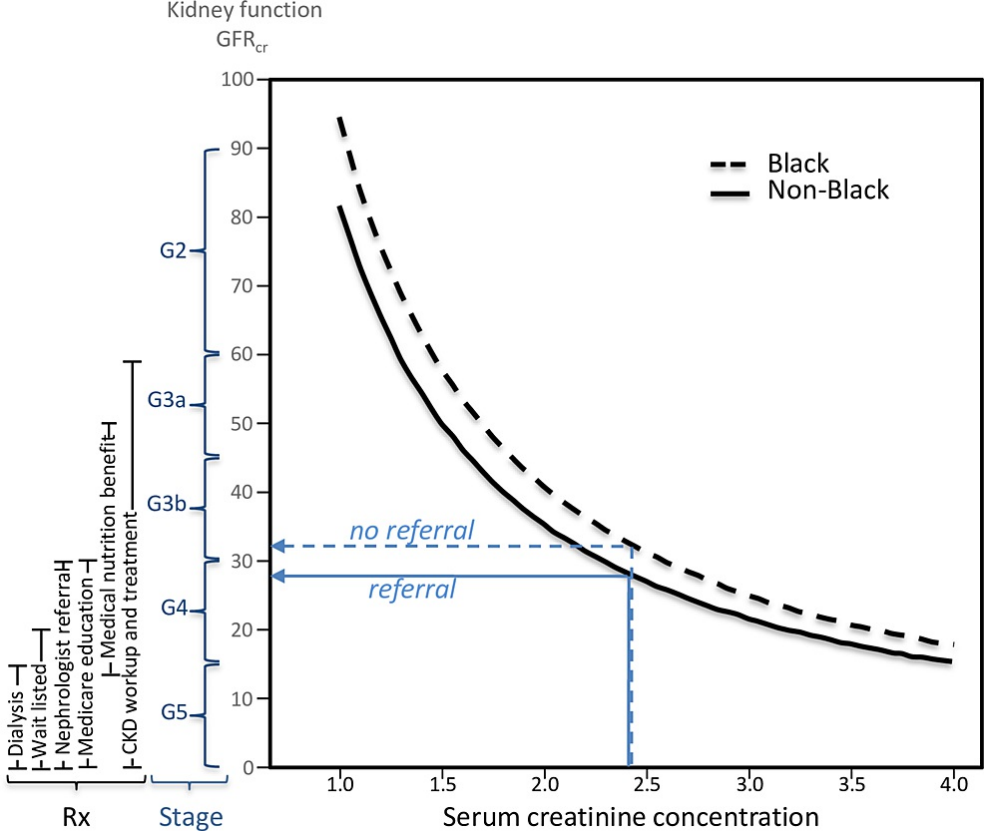
The relationship of serum creatinine concentrations to kidney function (mGFR) in Blacks and non-Blacks using the 2009 CKD-EPI formula for 60-year-old males Blacks and non-Blacks are assigned the same CKD stage and qualify for the same treatment when they have the same expected level of measured kidney function (GFR), which is not the same as having the same creatinine concentration.  Claims that Blacks are under-diagnosed or undertreated based on their creatinine concentration (which is not kidney function) ignore this fundamental principle.

The best overall measure of kidney function is glomerular filtration rate (GFR), i.e., the rate which plasma is filtered through the kidneys' functioning nephrons [1]. Chronic kidney disease (CKD) is defined as either a marker of kidney damage or GFR<60 mL/min/1.73m^2^ for three months. Among people with CKD, decreasing levels of GFR define stages of more severe CKD (i.e., GFR between 60-89, 45-59, 30-44, 15-29, and <15 mL/min/1.73m^2^ corresponding to CKD stage G2, G3a, G3b, G4, and G5, respectively [1]). The International Kidney Disease Improving Global Outcomes (KDIGO) guidelines recommend CKD workup and treatment at GFR <60, nephrologist referral at <30, and wait listing for preemptive kidney transplantation at ≤20 mL/min/1.73m^2^ (Figure 1) [2].

Although GFR is best determined by measuring the clearance of exogenous filtration markers directly (mGFR), it is more commonly estimated from serum concentrations of endogenous filtration markers (eGFR). One such endogenous marker is serum creatinine concentration, whose standardized measurements are widely available from high-throughput analyzers [1]. For over two decades, serum creatinine concentrations have been used for routine CKD screening and treatment, where higher concentrations are associated with lower (worse) GFR and greater (more severe) CKD. 

Self-identified Blacks in the United States (i.e., African Americans unless otherwise noted) have higher adult mean serum creatinine levels than non-Blacks independent of age, sex and mGFR [3,4]. Moreover, serum creatinine levels correlate positively with the degree of African ancestry, independent of income and education [3,5,6]. Therefore, race-correction factors have traditionally been included in the calculations of creatinine-based GFR estimates (eGFR_cr_, Appendix). Relative to non-Blacks, eGFR_cr_ in Blacks is increased by 21.2% in the 1999 Modification of Diet in Renal Disease (MDRD) formula [4] and by 15.9% in the 2009 Chronic Kidney Disease Epidemiology Collaboration (2009 CKD-EPI) formula [7]. Although the MDRD formula has been more widely used in North American clinical laboratories (>65%) [8], the 2009 CKD-EPI formula was specifically recommended by the international KDIGO organization [2]. 

Students and activists [9-13], concerned about the inclusion of race in the diagnosis and treatment of CKD and other conditions, initiated a debate on the role of race in medicine. They claim that Blacks are denied treatment for CKD because of the race correction factor. In deference to their concerns, and those of the congressional House Ways and Means Committee [14], the *National Kidney Foundation* and the *American Society of Nephrology* (NKF/ASN) Task Force on Reassessing the Inclusion of Race in Diagnosing Kidney Disease [15] removed race in CKD diagnosis and treatment guidelines [16], a decision seconded by the *National Kidney Foundation Kidney Disease Outcomes Quality Initiative* [17]. This decision affects over 10 million non-Blacks previously diagnosed with CKD whose disease severity will be administratively reduced, and one million Blacks not previously diagnosed or diagnosed with less-severe CKD being administratively advanced in CKD-disease severity [18]. This paper examines the arguments posed for excluding race correction as a prelude to discussing the Task Force’s decision. Specifically, it identifies that which was *not wrong* about the race-corrected 2009 eGFR_cr_ and that which is *not right* about the race neutral 2021 eGFR_cr_.

## Review

Rationale for excluding race corrections in estimating eGFR_cr_

A systematic literature review was used to identify the prevailing rationales for eliminating the race correction factor in the 2009 CKD-EPI and MDRD GFR_cr_ estimates. Twenty-three papers were identified among the 2,293 articles retrieved via the PubMed search criteria described by Braum et al. [19] and nine additional papers were retrieved among 2,419 additional articles identified via a PubMed search on “race” and “kidney disease” or “GFR.” Papers were initially chosen by a cursory reading of all titles and abstracts, followed by a full-text reading in triplicate of those appearing to qualify. In addition to the 32 qualifying papers, 21 additional papers were identified among the references cited in the 32 aforementioned articles and among the articles that cited the 32 aforementioned articles. This process yielded 18 reviews and other regular journal articles, 25 commentaries, and 11 editorials. The electronic search of the PubMed database was conducted on January 1, 2023 initially and periodically thereafter.

Although some papers are more circumspect [20-23], the systematic review identified fifty reviews, editorials, and commentaries in peer-reviewed journals [10,13,19,24-70] that argued for the abolition of race-based GFR_cr _estimation because Black race is: 1) a societal rather than biological construct [10,15,24-31,34-45,48-52,55,58,60-67,69,70]; 2) an imprecise proxy for genetic ancestry [13,24-26,29,31,43,44,46-50,60-65,67,70]; 3) poorly defined [10,13,19,34,39,43,44,48-52,55,62,63,67-69]; and 4) heterogeneous [10,26,29,30,34,35,44,46,52,66,67]. These articles also declared that the race correction: 5) lacked a physiological explanation [10,25-27,29-31,37,40,42,43,45,49,57,58,60,62,70]; 6) was invalid because it did not apply to non-US Blacks [10,25,29,30,31,44,46,50,52,63,66-69]; 7) was due to diet, sociological effects and racism [10,13,19,25,31,37,40,42,44,46,49,68]; 8) was inconsistent with the greater incidence of end-stage renal disease (ESRD) in Blacks vis-à-vis Whites [10,40,45,58]; 9) was originally based on inadequate non-representative samples [25,40,42,46,52,53,63,68]; 10) was an ecological fallacy [10,34,35]; 11) was negligible relative to the measurement precision of eGFR_cr_ [33,35,54,64]; and 12) was discriminatory because it denies CKD treatment to Blacks who would otherwise qualify [10,26,27-32,37-40,43,45,47,49-52,55-65,67-70]. Many asserted that the correction factor promotes racism [10,28,31,38,39,46,47,54,58,64,68,70], perpetuates the false belief that bodies or kidneys are biologically different in Blacks and non-Blacks [10,26,28,40,46,48,51,54,58,59,70], and violates the fundamental premise that racial health disparities are largely, if not exclusively, due to racism [24,30,32,34,37,40,43,47,56,63]. GFR_cr_ estimation in CKD diagnosis and treatment was singled out as the quintessential example of race-based medicine [39]. Arguments for the abolition of the race correction factor were often presented in the context of characterizing medicine per se as racist [10,24,26,39,47]. No published rebuttals to these specific arguments were identified, although several papers acknowledge the utility of race correction [20,22,71,72].

The *National Kidney Foundation/American Society of Nephrology Task Force on Reassessing the Inclusion of Race in Diagnosing Kidney Disease* removed race in CKD diagnosis and treatment guidelines [15], a decision potentially affecting the health care of over eleven-million patients [18]. The decision was justified on the basis that race is a societal rather than biological construct [15], although other arguments cited above no doubt contributed to the decision. Race correction was eliminated without ever challenging the validity of the assertions against it. The counterarguments that follow warrant consideration given the profound impact of the Task Force’s decision on so many lives. Nevertheless, the validity of these concerns in the context of CKD warrants careful scrutiny given the profound impact of the Task Force’s decision on so many lives.

Validity of the arguments for removing the race correction from the eGFR_cr_ formulas

Self-Reported Race Is a Societal Construct

The race corrections for MDRD and 2009 CKD-EPI were all based on self-reported or investigator-assigned race. The race-correction improves the prediction of mGFR regardless of whether “race” is a biological or social construct (p<<10^-16^) [16], and there is no statistical requirement regarding whether a covariate is biological or sociological. The legitimacy of age and sex as covariates but not race has no statistical foundation. Like race, biological age is imperfectly related to chronological age [73,74] and is affected by stress [75] and discrimination [76]. 

Self-Reported Race Is an Imprecise Proxy for Genetic Ancestry

Theoretically, measurement error in assigned ethnicity would be expected to attenuate its regression coefficient when predicting mGFR [77], suggesting the true effect of race is underestimated in these equations. In the United States, self-identified Whites are primarily of European ancestry (95.7%, with only 0.2% African ancestry [3]) and Blacks are primarily of African ancestry (82.6%, with only 15% European ancestry [3]). When whole-genome genotypes are used to characterize individuals’ continental ancestry, self-identified race/ethnicity is found to predict continental ancestry with greater than 96% accuracy [78]. 

Reported dose-response relationships between African ancestry and serum creatinine concentrations provide further evidence of race’s biological rather than sociological effect. These include Peralta et al.’s report of a significant ancestry-creatinine association in men beyond that explained by self-identified race [6], and Udler et al.’s report that each 10% increase in African ancestry increased creatinine concentrations by 1% in African Americans and 0.9% in Hispanic/Latino Americans [5]. Adjustment for African ancestry eliminated the significantly higher serum creatinine concentrations in self-identified Blacks than Whites in the Mount Sinai Medical Center (from P<0.001 to P>0.05) [5]

Blacks in the United Kingdom (UK) also have significantly higher creatinine levels than UK Whites. Analysis of the UK Biobank multiracial study cohort [79] suggests that the higher serum creatinine levels in Blacks are mostly attributable to their African ancestry. Specifically, adjusting for African ancestry in a multiple regression model largely eliminated the highly significant effect of Black ethnicity on serum creatinine levels (decreased from P<2x10^-16^ to a marginally significant P=0.05) whilst the highly significant effect of African ancestry on creatinine remained essentially unchanged when adjusted for Black ethnicity (P<2x10^-16^ before and after adjustment).

Self-Reported Race Is Poorly Defined

There is excellent agreement between provider-perceived and patient self-reported race in the Scientific Registry of Transplant Recipients data overall (95.3%), and in Blacks (98.6%), Whites (97.3%), Asians (99.0%), and Hispanics specifically (99.8%) [80]. Sensitivity to accurately classify non-Hispanic Whites and Blacks was reported to be ≥96% in 570,018 records from the Veterans Administration [81], ≥97% in 343,658 Medicare records [82], and 99% in 9170 cancer registry records [83]. Others also report strong agreement between self-identified and administratively assigned Black race (97.7% [84], 96.6% [85], 96% [86], 94% [87], 92% [88]). Concerns that mixed-race patients are not clearly assigned by 2009 CKD-EPI calculations [23,24,26,31,32,40,41,43-46,48,52,57,59,69] affect only 1.8% of the US population (i.e., respondents who identified as Black or African American in combination with another race group in the 2020 census [89]). Although race/ethnicity are less reliably reported by Hispanics (mostly ≥ 80% accuracy), Asians (mostly ≥75%), Native Americans (mostly≤56%), and Hawaiian/Pacific Islanders (30% and 38%) [90], these do not affect eGFR_cr_.

Blacks Are Heterogeneous

It appears contradictory to use Black heterogeneity and the lack of information on environmental factors to delegitimize 2009 CKD-EPI and MDRD race corrections [29,30] and then to combine non-Black and Black heterogeneity and ignore potential environmental effects in deriving the 2021 CKD-EPI eGFR_cr_ [15]. Although the effect of race cannot be identical for every individual, over the diversity of African ancestries and experiences that make up the US Black population as a whole, the race-corrected regression coefficients should theoretically provide the minimum variance unbiased estimate of log(GFR) vs. log(creatinine). Delanaye et al.’s claim that “skin color does not influence GFR or serum creatinine in a biological sense” [25] implies the existence of an argument never made. Similarly, a one-drop rule was never proposed for identifying Blacks in estimating eGFR_cr_ [48,54].

Race Correction Lacks a Physiological Explanation

Higher mean creatinine production in Blacks due to their greater mean muscle mass is a plausible biological explanation for their higher average serum creatinine concentrations vis-à-vis Whites. Many of the critiques attempt to undermine the race correction factor by challenging this explanation. A common misperception is that the race correction factor was derived from the theoretical effects of greater muscle mass in Blacks [19,30,31,47,53,59] when, in fact, the race correction factors were derived empirically to better estimate mGFR [4,7]. The physiological effect of muscle mass on circulating creatinine concentrations is consistent with the eGFR race-correction coefficient but it is not its source. 

Several lines of evidence suggest that the higher mean serum creatinine concentration in US Blacks is due to higher production: 1) higher mean creatinine concentrations in Blacks have been demonstrated in hemodialysis patients and in patients at the onset of uremia who are unlikely to have significant residual kidney function [91-94], and 2) the weekly amount of creatinine extracted by dialysis is about 40% higher in Black than White men when matched for age, weight, and dialysis dose [95]. 

Approximately 98% of circulating creatinine is derived from muscle [96]. Serum creatinine concentrations primarily reflect the balance between creatinine’s continuous production by muscle and excretion by glomerular filtration and to a lesser extent tubular secretion almost exclusively through urine [54]. Striated muscle mass correlates positively with total plasma creatinine and urinary creatinine excretion [97,98]. This association between muscle mass and creatinine production is consistent with the higher average serum creatinine concentrations in men vs. women [99], pre- vs. post-transfeminine and post- vs. pre-transmasculine patients [100], elite athletes vs. sedentary subjects [101], healthy patients vs. those with muscle wasting (cirrhotic, HIV, malignancy) [102], and in non-amputees vs. amputees [103]. Moreover, serum creatinine concentrations correlate negatively with muscle mass lost in amputees [103] and positively with lean body mass in non-amputees [104].

The higher average muscle mass in Blacks than non-Blacks is well established [105-119], and persists when adjusted for age, sex, height, and body weight [120,121]. The National Health and Nutrition Examination Survey (NHANES), a nationally representative cross-sectional study of health and nutritional status in the US, reported that muscle mass averaged 11% greater among Blacks than Whites, a difference similar in magnitude to the higher GFR_cr_ formula estimates assigned to Blacks [54]. Other studies report greater mean lean body mass (approximately three-fourths of which is skeletal muscle [122]) in Blacks than Whites [123,124]. The greater mean muscle mass in Blacks than Whites is consistent with the higher mean free testosterone concentrations in Blacks [125] and the established concordance between muscle mass and testosterone [126]. The correction factor does not posit systematically better kidney function in Blacks than non-Blacks [26,59,127], rather it posits that mean serum creatinine concentrations are greater in Blacks [25].

Critics of the 2009 CKD-EPI eGFR_cr_ claim that attributing the higher average creatinine concentrations in Blacks to their greater average muscle mass is “not scientifically sound” [10], “scientifically invalid” [42], not convincingly shown [25], cast in doubt [43], unsupported [27,26], “disproven” [31] debunked [58], invalid [62], “poorly substantiated” [37], “never proven by any reputable research“ [40], “not based on scientific data” [50], and lacking sufficient evidence [26]. Others assert the explanation is “a biological myth” [30], has “not been substantiated by rigorous scientific evidence” [57], that “no causal link has ever been confirmed [29], and represents “inappropriate racial stereotyping”[26]. These allegations are based on Hsu et al. report that statistical adjustment for muscle mass as estimated by bioimpedance did not eliminate the race-creatinine association [91]. However, inaccuracies in bioimpedance estimates of muscle mass [128] and incomplete adjustment for covariates measured with error [77] limit Hsu and colleagues’ conclusions.

Unenhanced computed tomographic (CT) imaging of the total lumbar muscle cross-sectional area (MCSA) taken at the middle of the third lumbar vertebra provides a surrogate marker of total body muscle mass [129]. Stehlé et al. reported that adjustment for MCSA between Black (African and Caribbean descendant) and White French accounted almost entirely for their differences in both urinary creatinine excretion and plasma creatinine levels [129]. Moreover, the smaller Black-White MCSA differences in females (117 vs. 111 cm^2^) than in males (193 vs. 173 cm^2^) corresponded to the to the smaller Black-White differences in plasma creatinine concentration (females: 66 vs. 61; males: 94 vs. 76 µmol/L) and urinary creatinine excretion (females: 11.0 vs. 10.2; males: 24.0 vs. 17.0 mmol/day). eGFR_cr_ and CT-measured MCSA were not significantly related to race, albeit the sample sizes were modest.

Race Correction Is Invalid Because It Does Not Apply to African Blacks

Another aspersion cast against the race correction factor is its inapplicability to African Blacks [25,29,31,46,67,130]. The greater accuracy of eGFR_cr_ without the correction factor in African Blacks does not necessarily discredit race adjustment in US Blacks, rather lower creatinine levels in African Blacks may simply place their eGFR_cr_ at or below US non-Black eGFR for any given mGFR. The overestimation of mGFR in Malawi, Ugandan, and South African Blacks by US-derived eGFR [131] is likely explained by the muscle mass of African Blacks being lower than that of US Blacks and more similar to non-Blacks in the United States. Specifically, lower fat-free mass (and presumably muscle mass which makes up approximately three-quarters of lean body mass) is reported for Blacks in Ghana (47.5 kg), South Africa (46.8 kg), Jamaica (49.2 kg), and Seychelles (48.6 kg) vis-à-vis the United States (58.6 kg) [132]. Fat-free mass and fat-free mass index are also reported to be lower in East African immigrants than African-American girls (both P = 0.002) [133]. Eastwood et al. [134] reported that mGFR by creatinine clearance in Ashanti (average BMI of 21.1 kg/m^2^) was more similar to 2009 CKD-EPI without race correction. Bukabau et al. reported that mGFR by plasma iohexol clearance in Congolese (average BMI of 23.5 kg/m^2^) was also more similar to 2009 CKD-EPI without race correction [135]. Again, the substantially lower BMI in Ashanti and Congolese than in US Blacks is likely to reflect their lower muscle mass. The need for formulas that estimate eGFR_cr_ to be population-specific [61] is formally recognized in the European Kidney Function Consortium (EKFC) equation [136].

Race Correction Is Due to Diet, Sociological Effects, and Racism

Higher serum creatinine concentrations can also be due to increased muscle mass from habitually high protein intake and postprandially following cooked meat intake (particularly beef) [137]. However, Blacks report being significantly less likely to consume excessive protein (≥0.8 g/kg/day) than Whites with CKD in NHANES [138], consume significantly less protein on average than Whites in the Chronic Renal Insufficiency Cohort (CRIC) study [139], and to consume similar total protein as Whites for the US population as a whole [140]. Annual per capita intake is slightly less in Blacks than Whites for both total meat (35.6 vs. 37.5 kg/person) and beef (20.2 vs. 22.1 kg/person) [141]. When adjusted for measured GFR, Blacks had higher serum creatinine concentrations despite lower protein intake than non-Blacks in the MDRD [142] and CRIC studies [3,142]. (Left unexplained is the higher estimated net acid excretion in Blacks than Whites [143], which could reflect higher animal protein intake in the former.)

Poverty, low education, and underemployment are associated with CKD and microalbuminuria [144]. Contrary to the assertion that Black-White health disparities are due to social (not biological) factors, meta-analyses show that the absolute risk of CKD is higher in American Blacks than Whites, independent of whether African Americans have low socioeconomic status (SES) or high SES [145]. Moreover, the effects of low SES on CKD and ESRD risk are actually 30% smaller in Black than in White Americans [145]. Prospectively, the Jackson Heart Study showed that cumulative lifetime SES was unrelated to incident CKD and annual eGFR_cr_ decline in Black Americans [146]. Moreover, SES (i.e., deprivation) was also unrelated to serum creatinine levels in the UK Biobank multiracial study cohort [79]. Similarly, adjustment for social determinants of health did not substantially change the 16% and 10% higher mean creatinine concentrations in Blacks than non-Blacks in the MDRD and CIRC studies, respectively, when adjusted to the same mGFR [142], nor the 21% higher mGFR in Blacks than non-Blacks in the MDRD study nor the 13% higher mGFR in the CRIC when adjusted to the same serum creatinine concentration [142].

There is little published evidence showing discrimination and stress decreases kidney function. Everyday discrimination was low and showed no concordance with CKD in Caribbean Blacks residing in the United States [147], or Ghanaians living in Europe [148]. There was also no significant cross-sectional association between eGFR_cr_ and the “Experience of Discrimination” scale in the biracial Healthy Aging in Neighborhoods of Diversity Across the LifeSpan study [149]. The Jackson Heart Study actually reported that greater life stressors were associated with a lower CKD prevalence [150], whereas others report no association of stress with eGFR_cr_ in Blacks [151]. Prospectively, psychosocial factors were not associated with eGFR_cr_ decline or incident CKD in the 3,390 Jackson Heart Study Blacks during eight years of follow-up [150]. The reported inverse association between greater everyday discrimination (i.e., the type that is common in older adults) and eGFR in the Health and Retirement Study pertains to a sample that was 83% White and 8% Black and is therefore unlikely to be specific to racial discrimination, particularly given that the effect was not different by race [75]. Speculation that the elevated risks of CKD or ESRD in Blacks are due to weathering [34,42], discrimination-induced allostatic load [10,42], or the epigenetic transgenerational transmission of the effects of racism [10] requires empirical evidence. Whether the 2.4 mL/min/1.73m^2^ effect of racial discrimination on eGFR_cr_ in young and middle-aged Brazilians [152] is relevant to US Blacks is not known. 

The Race Correction Is Inconsistent With the Greater Incidence of ESRD in Blacks Vis-à-Vis Whites

Despite the protestation that health disparities are sociological [24,40,47], biology may primarily underlie racial differences in CKD progression and ESRD. The genetic risk of ESRD in Blacks may explain much of the so-called paradox between the Black’s higher prevalence of ESRD despite their higher average eGFR_cr_ [10,19,58]. 

Approximately 70% of the excess risk of development, progression, and severity of CKD in Blacks is attributable to any combination of the G1 or G2 risk alleles of *APOL1*, the gene encoding apolipoprotein L1 [153,154]. More than half of American Blacks carry at least one risk variant, and the 13% (5 million) of Blacks that carry the high-risk genotype (2 alleles) have at least a 15% lifetime risk of kidney disease [155]. *APOL1* renal risk variants are not present on European or Asian chromosomes [156]. Adjustment for African ancestry eliminates the significantly higher rate of ESRD in Blacks than non-Blacks, due largely to the effects of the *APOL1* genotypes [5]. The significantly higher risk of kidney failure in Blacks than non-Blacks is limited to Blacks with high-risk *APOL1* genotype [157], as is the significantly faster rate of eGFR_cr_ decline in Blacks than Whites [158].

In addition, approximately 8% to 9% of American Blacks carry one copy of the sickle hemoglobin gene, which is associated with a faster decline in eGFR compared to patients with a normal hemoglobin phenotype [159]. The decline is even more rapid in those with sickle cell disease [159]. Despite having greater delays in nephrology referral, being less likely to receive home or peritoneal dialysis or undergo arteriovenous fistula placement, and increased infection during peritoneal dialysis [10], Blacks experience less mortality than White dialysis patients (136 vs. 207 deaths per 1,000 patient years [160]) and 19% lower odds of mortality than rural White ESRD patients [161]. This may be due in part to muscle mass: 1) being greater on average in Blacks than Whites, and 2) predicting greater longevity in CKD patients [162,163]. 

Statistical Issues (i.e., Race Correction Based on Small Unrepresentative Samples Representing an Ecological Fallacy and Having Negligible Effect)

The standard errors for the race correction factors reflect the uncertainty associated with the small sample size in earlier papers [4,7], which have since been confirmed in larger studies [16]. Race correction factors were estimated from regression analyses of individual data, they are not examples of ecology fallacy, i.e., the “flawed inference of individual characteristics based on group data” [10]. Those who argue that the race correction is negligible relative to the measurement precision of eGFR [33,54] ignore the statistical fact that measurement error can be reduced by averaging over multiple samples whereas the bias introduced by excluding the race correction factor will remain constant. 

The Race Correction Is Discriminatory Because It Denies CKD Treatment to Blacks Who Would Otherwise Qualify

mGFR is the gold standard for diagnosing CKD. CKD diagnosis and treatment recommendations should be the same for Black and non-Black patients at identically measured GFR. The original race-based eGFR_cr_ formula (2009 CKD-EPI, eq 2) provides the best approximations to their common mGFR. Thus identical 2009 CKD-EPI eGFR_cr_ values should lead to identical treatments in Blacks and non-Blacks with respect to their mGFR (Figure 1), which does not necessarily mean identical treatment by serum creatinine concentrations. Requiring identical treatment for Blacks and non-Blacks having the same creatinine concentrations is tantamount to asserting Blacks should be diagnosed, staged, and treated for CKD at higher mGFR than non-Blacks. Other than one notable exception [164], this simple explanation for the race adjustment never appears in the medical or popular press, nor is it recognized in recent efforts to criminalize treatment algorithms that adjust for race [165,166]. Assigning CKD diagnosis and treatment based on the best estimate of mGFR is the driving principle behind MDRD, 2009 CKD-EPI, cystatin C, β2-microglobulin and β-trace protein formula (albeit imperfectly applied when significant race coefficients are excluded [167]). Claims of discrimination based on different eGFR_cr_ for clinically identical age, sex, and creatinine concentrations in Blacks and non-Blacks ignore this driving principle [24,38,40,50,68]. 

Comparison With Other CKD Markers

Marzinke et al. [67] identified discordance between eGFR and other markers of kidney disease in Blacks that could argue against the race coefficient. This included: 1) increasing Black-White parathyroid differences with decreasing eGFR_cr_ [168]; 2) more frequent anemia, hyperuricemia, and hyperparathyroidism in Blacks than Whites with eGFR_cr_<60 mL/min/1.73m^2^ [169]; and 3) greater prevalence of albuminuria and hyperuricemia Blacks than Whites with eGFR_cr_ between 60 and 80 mL/min/173m^2^ [170]. Others have shown that higher concentrations of parathyroid hormone in Blacks than White for all stages of CKD [171]. If these are due to Black kidney function being overestimated by the race correction factor, then this would imply that parathyroid hormone concentrations, anemia, hyperuricemia and albuminuria are all affected at higher mGFR in Blacks than Whites. In addition, Marzinke et al. [67] reported that eGFR_cr_ overestimated mGFR in two studies of US Black donors [172,173] as well as third study of Afro-Caribbean Blacks in the UK with eGFR_cr_>90 as estimated by MDRD [174]. They also cited Mahmud et al. [172] report that the association between lower eGFR_cr_ and higher rates of acute kidney injury (AKI) in cirrhotic patients was stronger without the race correction. Mathematically, shifting the higher risk Blacks to the lower range of eGFR_cr_ would indeed strengthen the association. Albeit thought-provoking, none of Marzinke et al. observations overturn Levey et al.'s [7] more direct derivation of the race correction based on a developmental data set of 8,254 participants in 10 studies and validation data set of 3,896 participants in 16 studies.

Race-free estimation of eGFR

The criticisms pertaining to defining race would be more apropos if the goal was to characterize and quantify racial differences in serum creatinine concentrations (i.e., definition, genetic ancestry, heterogeneity, confounding factors, biological basis), but it is not. The goal is to best estimate mGFR in a clinical setting on the basis of biomarkers, sex, age, and patient self-report, including self-reported or clinically assessed race. The empirically derived race-correction factors adjust for non-GFR effects on serum creatinine concentrations associated with race, which could include race-related differences in stress, poverty, medications, chronic illness, nutritional status, diet, and creatinine tubular secretion. The race correction is highly significant (P<<10^-16^) and is mathematically required by the higher average serum creatinine concentrations in US Blacks than Whites in the absence of any Black-White difference in average mGFR. The race-correction improves the prediction of mGFR regardless of whether “race” is a biological or social construct, and there is no statistical requirement regarding whether a covariate is a biological or sociological. 

**Figure 2:**
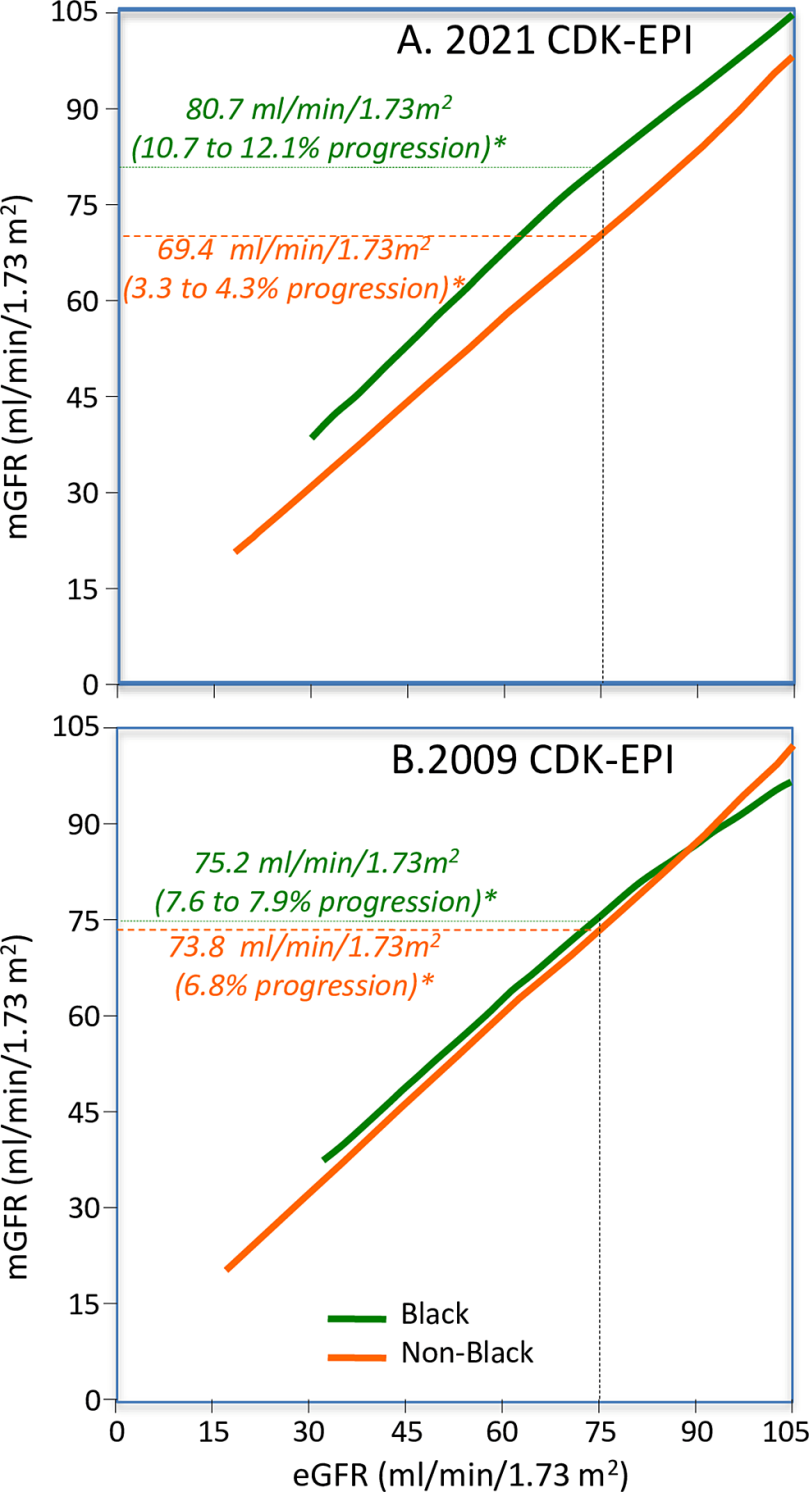
Relationships of mGFR to (A) race-free (2021) CKD-EPI eGFR and (B) 2009 race-corrected CKD-EPI eGFR. Whereas a particular eGFR (e.g., 75 mL/min/1.73m^2^) correspond to essentially the same mGFR in Blacks and non-Blacks when estimated by 2009 CKD-EPI (A), it corresponds to very different mGFR in Blacks than non-Blacks when estimated by 2021 CKD-EPI eGFR (B). Adapted from Inker et al.’s Figure 1 [16].

The NKF/ASN Task Force chose to recommend a revision of the 2009 CKD-EPI eGFR prediction formula that does not include the race as an explanatory variable [15]. This refit formula, designated as “2021 CKD-EPI eGFR” (Appendix) was derived from Inker et al.’s statistical analyses of age, sex, race and serum creatinine concentrations as predictors of mGFR in the 8,254-patient developmental dataset and a 4050-patient validation dataset [16]. Bias, calculated in the current report as the eGFR-mGFR difference, was used to assess whether eGFR underestimated (negative bias) or overestimated (positive bias) mGFR. Revised eGFR estimates were derived with (Figure 2B) and without including race as an explanatory variable (Figure 2A). Excluding race in the derivation of eGFR changed the median bias from +3.7 to -3.6 mL/min/1.73m^2^ in Blacks and from +0.5 to +3.9 in non-Blacks, i.e., promoting CKD diagnosis in Blacks at the cost of restricting diagnosis in non-Blacks. Their decision was driven not by scientific discussions of the clinical utility of self-identified race, but rather the statistically irrelevant claim that race is a societal rather than biological construct [15]. The exclusion of race was a pre-condition to the choice of formulas and not the consequence of rigorous statistical evaluation [15]. In their deliberations, the Task Force and commentators ignore the overriding fact that mGFR is the gold standard for diagnosing CKD.

Rush to implement

The push by the Task Force for the immediate adoption of their race-free eGFR through clinical laboratories precluded any debate on their decision. Race-free eGFR formula have been mandated by the Veterans Administration since April 2022 [176] and by the US Organ Procurement and Transplantation Network (OPTN) since July 2022 [177]. As of March 2022, 30% of clinical laboratories report using the race-free 2021 CKD-EPI equation and an additional 18% expect to be added by the end of the year [178]. In fact, the conversion to 2021 CKD-EPI by Quest Diagnostics since July 2022 and by LabCorp since February 2022 alone accounts for over 40% of the US test volume [179]. The Epic Cosmos database of 167 million US patients and 247,000 physicians report that 2021 CKD-EPI was used in 70% of recent laboratory reports as of late October 2022 [179].

Sample distortion

Blacks were overrepresented in the 2009 CKD-EPI developmental and internal validation data sets (31.3%) relative to their proportion in the United States (13.4%) for good reason. Black enrichment provided a more precise estimate of the race correction factor in estimating eGFR_cr_. However, its retention in calculating 2021 CKD-EPI eGFR purportedly served to distribute the absolute bias equally by race (racial equity) rather than by population percentage (individual equality) [18]. Inker et al. (Figure 5S and 9S in reference [16]) demonstrated how weights might be chosen to manipulate the distribution of bias. To this end they rationalized that the weights approximated the national burden of kidney failure among Black adults (37%) rather than their share of the general population (13%) [18]. 

Blacks and Whites re-classified

**Figure 3:**
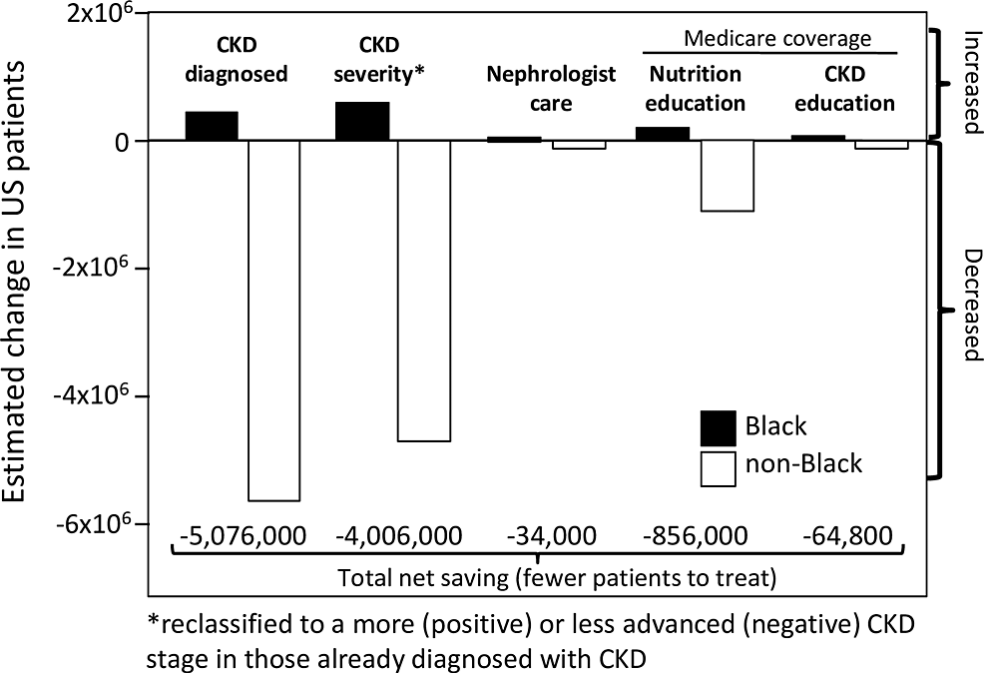
Estimated effect of changing the calculation of eGFR from the race-corrected 2009 CKD-EPI formula to the refit race-free 2021 CKD-EPI formula, showing much greater impact on treatment in non-Blacks than Blacks An additional enticement to adopting 2021 CKD-EPI formula is the reduced patient load. Determined from 44,360 participants of the 2001-2018 National Health and Nutrition Examination Survey weighted to represent the adult US population (adapted from data presented in reference [18]).  CKD staging and treatment corresponds to the 2012 Kidney Disease: Improving Global Outcomes (KDIGO) Clinical Practice Guideline for the Evaluation and Management of CKD [2]. CKD defined as eGFR<60 mL/min/1.73m^2^ or urine albumin-creatinine ratio>30 mg/g without the chronicity requirement.

Application of the old (2009 CKD-EPI) and new revised formula (2021 CKD-EPI) to 44,360 NHANES participants suggests that the race-free equation negates CKD in 5.51 million non-Black adults, and reclassifies CKD to a less advanced stages in an additional 4.59 million non-Black adults in the name of racial equity (Figure 3) [18]. Specifically, 434,000 Blacks would become newly CKD diagnosed while 584,000 Blacks would be shifted to more advanced CKD [18]. Among veterans, the revised formula would shift 289,242 non-Black veterans from CKD stages 3 to 4 to less-severe CKD (i.e., eGFR ≥60 mL/min/1.73m^2^) in order that 66,190 Black veterans qualify for G3-G5 CKD [180]. Among University of Washington Medicine patients, CKD in 3582 non-Blacks would be ignored so that 272 Blacks could receive a CKD diagnosis of eGFR<60, and 1765 additional non-Black CKD patients would be shifted to a less-severe disease stage so that an additional 128 Black CKD patients could have their disease severity increased [181]. As an additional incentive, the additional Black patients being referred to and cared for by nephrology would be more than made up for by the decrease in non-Blacks with eGFR<60 mL/min/1.73m^2^, leading to fewer referrals overall [130]. Adjustment to a less severe CKD in non-Blacks could result in inappropriately prescribing or overdosing common medications (metformin, gabapentin, tramadol, atenolol, rosuvastatin, ciprofloxacin [182]) in 1.47 million non-Blacks in the US [18].

Misclassified CKD

It is unlikely that statistical biases introduced by excluding the significant race correction factor would reveal heretofore-undiagnosed CKD in Blacks while excluding incorrectly diagnosed CKD in non-Blacks. Nevertheless, in their quest to justify race-free eGFR estimation, many credit the 2021 CKD-EPI revision with being able to classify CKD better than mGFR per se [53]. For example, Diamantidis et al. explained that “the use of a race coefficient likely masks real differences in CKD prevalence and hinders opportunities for timely identification and clinical management to slow CKD progression among Black enrollees” [183]. Johansen and Powe claim it is not possible to know whether the 2021 reclassification is not more accurate than the 2009 classification [184], referring to the 2021 USRDS [185] statement that “Use of an estimating equation [2009 CKD-EPI] that leads to a higher eGFR_cr_ among Black patients may be masking disparities in CKD prevalence earlier in the course of disease.” The US Organ Procurement and Transplantation Network (OPTN) asserted that by prohibiting the Black race coefficient from use in eGFR calculations “Black kidney candidates’ eGFR values will be more reflective of their actual kidney function” [186,187].

The original 2009 EPI-CKD eGFR has had a long history of acceptance by the international KDIGO community, but this may change for the 2021 refit. The 2021 CKD-EPI eGFR formula: reclassifies 9.9% of the total population and 36.2% of the G3-G5 CKD population to a lower (less severe) CKD category in Swedes [188], produces a 25% decrease in CKD prevalence in Danes [189], reclassifies 20% of stages 3-5 CKD and 10% of stages 4-5 CKD to less severe CKD in Welsh [190], and reclassifies 15% of the total population and 27% of stages 3-5 CKD population to less severe CKD categories in Spaniards [191]. The new eGFR formula overestimates mGFR: by over four fold as much as the original formula (median bias 3.6 vs. 0.8 mL/min/1.73m^2^) in French [192], by ≥two-fold (median bias 5.7 vs. 2.4 [192], 5.5 vs. 2.4 mL/min/1.73 m^2^ [193], 7.40 vs. 3.96 mL/min/1.73m^2^ (7) [194]) in Europeans, by two-fold (median bias 6.4 vs. 3.3 mL/min/1.73m^2^) in Chinese [195] and by nearly three-fold (median bias 4.8 vs. 1.8 mL/min/1.73m^2^) in Koreans [196]. The race-free 2021 CKD-EPI overestimated mGFR by 3.22 vs. 0.30 mL/min/1.73m^2^ by the original formula in Parisian Whites while underestimating mGFR by 5.09 mL/min/1.73m^2^ in Parisian Blacks [194]. The original 2009 CKD-EPI provided an essentially unbiased estimate of mGFR in Parisian Blacks (median bias 0.24 mL/min/1.73m^2^) [194]. Analyses of the UK Biobank data show that 3463 fewer non-Black British in their sample would be classified as CKD G3-5 (33.9% decrease) so that 113 additional British Blacks would be classified as CKD G3-5 (90% increase) [190].

**Figure 4:**
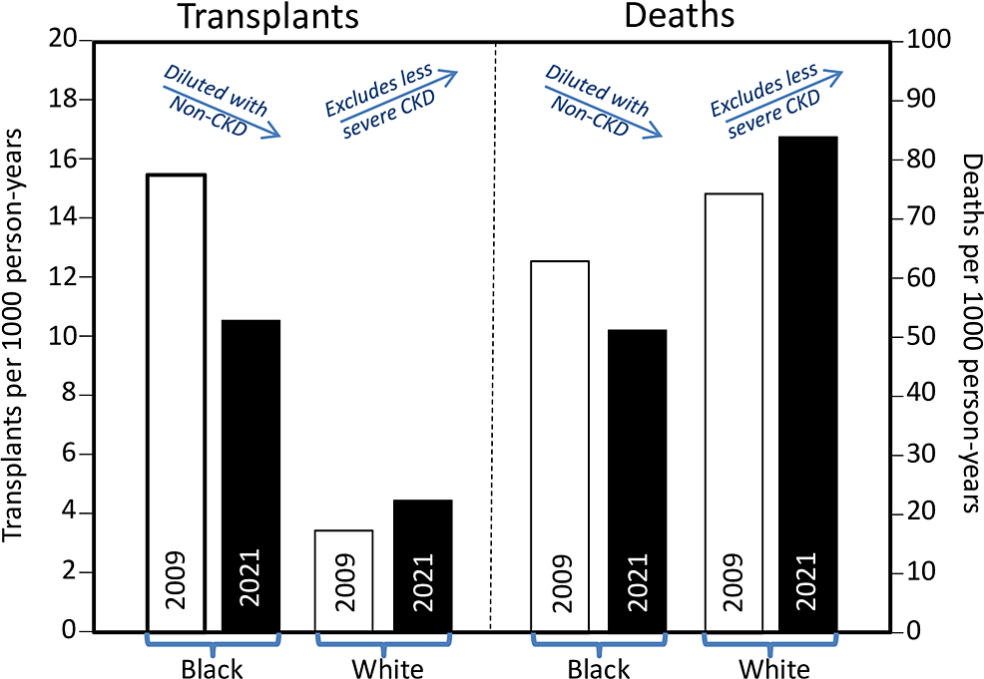
The 2021 CKD-EPI eGFR misclassifies healthy Blacks to ≥ stage 3 CKD and misclassifies ≥3 stage Whites to being CKD-free when transplantation and death are used as proxies for mGFR Results are contrary to claims that the 2021 CKD-EPI reveals previously undetected CKD in Blacks [53,180-184]. The difference between and 2009 and 2021 CKD-EPI eGFR is due to the change the bias from +3.7 to -3.6 mL/min/1.73m^2^ in Blacks and from +0.5 to +3.9 in non-Blacks. Based on data collected by the US Veterans Health Administration (VHA) between January 2007 and December 2016 in 101,693 Black and 449,802 White veterans classified as ≥stage 3 according to the 2021 CKD-EPI and 84,090 Black and 507,303 White veterans classified as ≥stage 3 according to the 2009 CKD-EPI (adapted from data presented in [197]).

Yan et al. [197] provide direct evidence that 2021 CKD-EPI does not reveal heretofore-undiagnosed CKD in Blacks. This becomes evident when the five-year prospective risks for kidney failure replacement therapy (KFRT) and mortality are interpreted as proxies of baseline CKD. If the Blacks added by 2021 CKD-EPI are representative of those qualified by the original 2009 CKD-EPI, then the additional Blacks should minimally affect KFRT and mortality risk. In fact, Figure 4 shows substantially lower risks for KFRT (10.5 vs. 15.2 per 1,000 patient years) and death (51.6 vs. 62.9 deaths per 1,000 patient years) when incident GFR category 3 or higher is based on the race-free 2021-CKD-EPI than the race-corrected 2009 CKD-EPI eGFR_cr_ [197]. In contrast, the reassignment of non-Blacks with CKD to disease-free status leaves only the more severely affected Whites as evident by the higher risks for KFRT (4.4 vs. 3.4 replacements per 1,000 patient years) and death (83.5 vs. 74.3 deaths per 1,000 patient years) when incident CKD is based on the race-free 2021 CKD-EPI eGFR_cr_ [197].

Using KFRT and mortality as proxies of baseline CKD, Gutiérrez et al. data also suggest 2021 CKD-EPI identifies CKD more-poorly than 2009 CKD-EPI [198]. Compared to the relatively unbiased 2009 CKD-EPI eGFR_cr_ (Figure 2B), the 2021 CKD-EPI inclusion of negatively biased eGFR_cr_ in Blacks and positively biased eGFR_cr_ in non-Blacks (Figure 2A). Using the 2021 instead of the 2009 eGFR_cr_ eliminated the significantly greater Black risk for KFRT at both 60 (i.e., significant 2.8 vis-à-vis nonsignificant 1.30 hazard ratio) and 30 mL/min/1.73m^2^ (i.e., significant 1.6 vis-à-vis nonsignificant 1.3 hazard ratio), and the significantly greater Black risks for all-cause mortality (i.e., significant 1.2 vis-à-vis nonsignificant 1.0 hazard ratio) and cardiovascular mortality (i.e., significant 1.4 vis-à-vis nonsignificant 1.1 hazard ratio) at 60 mL/min/1.73m^2^.

Similarly, 28,966 person-years of follow-up of northeastern Italians (Caucasian) in the Initiative on Nephropathy (INCIPE) Study showed that patients who were reclassified from CKD by 2009 CKD-EPI to non-CKD by the 2021 CKD-EPI equation had a mortality rate (35.7 per 100 person-years) much closer to patients consistently classified as CKD (33.0 per 100 person-years) than to patients consistently classified as non-CKD (5.8 per 100 person-years) [199]. Another study by Fu et al. [188] showed that predominately White Swedes reclassified to stage G2 from stage G3a CKD by the race-free 2021 CKD-EPI were more similar to Swedes consistently classified as stage 3a CKD than stage 2 CKD with respect to their prevalence of hypertension (44.7% closer to 49.8% than 24.7%), diabetes (13.7% closer to 16.0% than 7.0%) and cardiovascular disease (38.8% closer to 45.2% than 18.5%), suggesting that their original 2009 CKD-EPI classification was more correct. Moreover, during 9.5 years of follow-up, the 2009 formula was more predictive than the 2021 CKD-EPI formula for all-cause mortality (hazard ratio: 38.8 vs. 27.0 for eGFR_cr_ of 15 vs. 95 mL/min/1.73m^2^), cardiovascular mortality (81.8 vs. 49.4), and major cardiovascular events (31.5 vs. 23.5) [188]. Discrimination for the Kidney Failure Risk Equation was identical when using the 2021 or 2009 equations, however.

Finally, we consider Muiru et al.’s [200] recent claim to provide empirical evidence that the race-free 2021 CKD-EPI eGFR_cr_ unmasked Blacks who were at high-risk of CKD progression over five years. When eGFR_cr_ was calculated using 2009 CKD-EPI equation, they showed that: a) stage 3 Blacks had a higher risk of progressing to more severe CKD than Whites, whereas b) stages 1 and 2 Blacks had a similar risk of progressing to more severe CKD as Whites. In contrast, when eGFR_cr_ was calculated using 2021 CKD-EPI, Blacks had a higher risk of disease progression than Whites across all baseline CKD stages. The additional stages 1 and 2 Blacks progressing to more severe CKD were purported to have been unmasked by 2021 CKD-EPI.

Muiru et al.’s claim is problematic because the differential bias (eGFR-mGFR) between Blacks and Whites confounds Black-White disease comparisons. Specifically, when Blacks and Whites are compared using 2009 CKD-EPI eGFR_cr_ they are compared at the same mGFR (Figure 2B), but when they are compared using 2021 CKD-EPI eGFR_cr_ they are compared at different mGFR (apples and oranges, Figure 2A). Within each CKD stage, mGFR will therefore be higher in Blacks than Whites when calculated by 2021 but not 2009 CKD-EPI. Consistent with other reports [201], Muiru et al.’s paper showed that the five-year probabilities of transitioning to a higher stage were consistently greater for patients who were stage 1 than stage 2 at baseline, suggesting that within this range, the transition probability is greater at higher mGFR. Therefore, for patients classified as stages 1 and 2 at baseline by 2021 CKD-EPI, the Blacks’ higher mGFR is expected to produce a greater apparent probability of disease progression than the Whites’ lower mGFR. This suggests that acknowledged bias explains the greater transition probability in Blacks than Whites when classified at baseline by in 2021 CKD-EPI. (The differential bias may also contribute in part to the Black-White differences in progression reported by Choi et al. [201].)

Exaggerating health disparities

**Figure 5:**
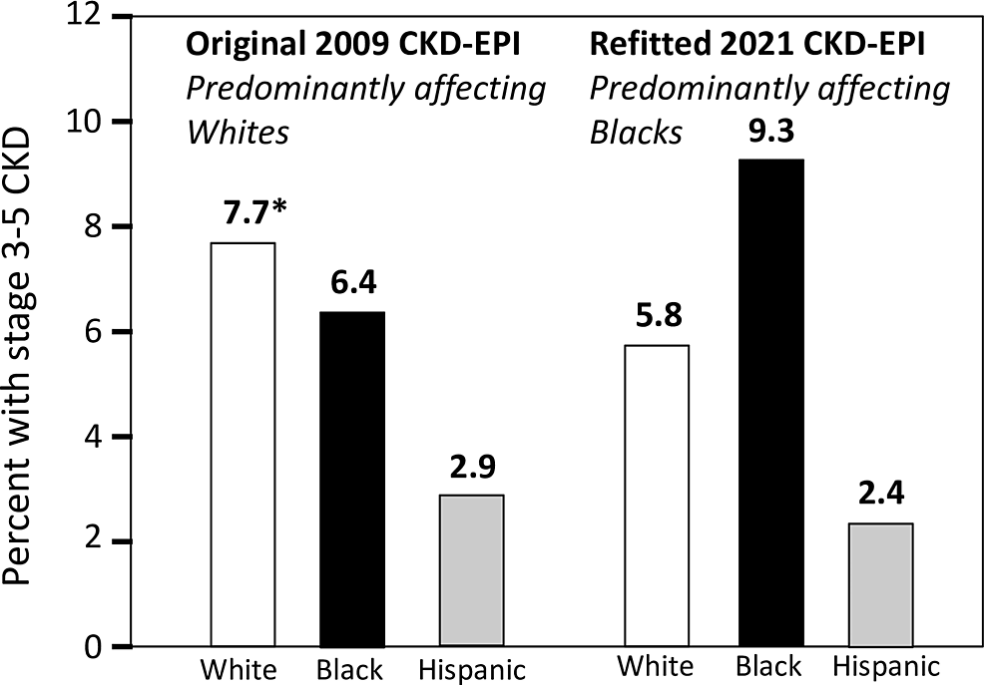
Exaggerated health disparities produced by biases in the refitted 2021 CKD-EPI equation vis-à-vis the 2009 CKD-EPI equation. The bars depict the prevalence of stages 3-5 CKD in 2015-2018 NHANES participant ≥18 years old (adapted from data provided in the Figure 14.1a of the 2021 USRDS [185]). Whereas the 2009 CKD-EPI equation attributed the greatest CKD burden to Whites, the 2021 CKD-EPI equation attributed the greatest burden to Blacks.

The 2020 United States Renal Data System (USRDS, their Figure 1.1 in reference [202]) reported that the prevalence of Grate 3 to 5 CKD was greater in non-Hispanic Whites than Blacks prior to the revision, i.e., 8.6% vs. 5.6% between 2003-2006, 7.5% vs. 5.8% for 2007-2010, 8.5% vs. 6.1% for 2011-2014, and 8.4% vs. 6.6% for 2015-2018, but this did not fit the narrative. By using the revised eGFR formula (2021 CKD-EPI), Figure 5 shows that CKD stages 3-5 was transformed from a disease affecting Blacks and Whites similarly (6.4% vs. 7.7%, respectively from 2015 to 2018 NHANES) into a disease that disproportionately affected Blacks (prevalence 9.3% vs. 5.8% in non-Hispanic Whites, derived from Figure 14.1a in reference [185]). This transformation is entirely attributable to the 2021 CKD-EPI equation’s negative bias for Blacks, which increased their stages G3-G5 CKD prevalence by 45%, and the equation’s positive bias for non-Blacks that reduced their prevalence by 25% [185]. As a result of this manipulation, the prevalence of stages G3-G5 CKD were substantially greater in Blacks than White in 2005-2008 (7.9% vs. 5.8%), 2009-2012 (8.5% vs. 5.9%), 2013-2016 (8.4% vs. 6.3%), and 2017-2020 (9.1% vs. 6.3% Figure 1.2 in reference [203]). The Centers for Disease Control (CDC) chose to report that the prevalence of stages 1-4 CKD was greater in Blacks than Whites (16.3 vs. 12.7% [204]) presumably using the un-credited CKD-EPI 2021 equation rather than the equal rates (15.7 vs. 15.6%, Figure 1 from reference [202]) using 2009 CKD-EPI in 2015-2018 NHANES participants. Similarly, the National Kidney Foundation claim that Blacks represent a disproportionate number of the 37 million US adults with CKD based on 2021 CKD-EPI equation [205]. Gutiérrez et al.'s analyses of 62,011 participants from eight US-based cohorts showed the 2021 revision increased the Black vs. non-Black prevalence ratio of CKD stages 3-5 from nonsignificant 0.98 (11% vs. 12%) to a highly significant 1.8 (15% vs. 9%) and increased the prevalence ratio of ≥ stage G4 CKD from 2.5 (1.7% vs. 0.7%) to 3.8 (1.9% vs. 0.52%) [198]. Schneider and Schneider analysis of 459,518 non-Black and 7,295 Black subjects in the UK Biobank cohort also showed that 2021 CKD-EPI changed the prevalence of CKD stages 3-5 from affecting primarily non-Blacks (2.4% vs. 1.7% in Blacks) to affecting primarily Blacks (3.3% vs. 1.6% in non-Blacks) [206].

Unequal CKD treatment

Differential Treatment

There is no evidence to suggest that the 10 million re-classified non-Black patients have been overtreated in the US. In fact, multiple sources show that Whites have been systematically undertreated for CKD vis-à-vis US Blacks even before the introduction of race-neutral eGFR_cr_. 

In cross-sectional study of 452,238 CKD patients not requiring dialysis who are actively engaged in medical care, Chu et al. [207] found that Blacks reported higher angiotensin-converting enzyme inhibitor (ACEi) and angiotensin II receptor blocker (ARB) use than Whites (76.7% vs. 72.3% in 2018-2019), higher statin use (69.1% vs. 61.5% for Whites), higher nephrology care (72.9% vs. 58.3% for Whites), and higher albuminuria testing (41.0% vs. 30.7% for Whites). Similarly, CKD epidemiologic surveillance data from the Veterans Affairs Health System [208] showed non-Hispanic Black veterans were more likely to have filled ACEi or ARB prescriptions, seen nephrologists (among patients with an eGFR <30 mL/min/1.73m^2^), and undergone urine albumin-creatinine ratio testing than White veterans. Lee et al. [209] reported that the odds for hypertensive, non-diabetic patients being tested for urine albumin-to-creatinine ratio within one year was 19% greater in Black and other non-White patients than in Whites. Gao et al. [210] reported that Black CKD patients in a Department of Defense Health System were significantly more likely to be seen by nephrology than White patients (28% versus 14% if stage 3 and 76% vs. 64% if stage 4), and were significantly more likely to be prescribed ACEi or ARB. Among 2019 Medicare beneficiaries with CKD, Blacks were more likely to use ACEi and ARB (63.1% vs. 60.1% in Whites), potassium binders (1.9% vs. 1.2%), and phosphorus binders (0.7% vs. 0.4%) and receive outpatient nephrology visits (1.2 vs. 0.9 per person-year) [182]. Among Partners Healthcare patients, Blacks were more likely to be co-managed by a nephrologist than Whites (15% vs. 8.6%), particularly for stage G3 CKD [211]. Greater nephrology care has also reported for Black than White veterans with advanced CKD (52.4% vs. 34.6%) [212]. The NHANES study also showed 40% greater odds for ACEi and ARB use in Blacks than Whites [213]. A review of electronic health record data showed nephrology referrals were significantly greater in Black than White primary care patients in the Saint Louis metropolitan area [214] and in Triad and western North Carolina [215]. 

Sodium/Glucose Cotransporter 2 Inhibitor (SGLT2i) and Glucagon-Like Peptide 1 Receptor Agonists (GLP-1RA) Treatment

Rather than finessing eGFR calculations, greater health benefits may be achieved by preventing CKD progression in all patients through newer glucose-lowering medications, including SGLT2i, which has been shown to reduce kidney disease progression and mortality in CKD patients regardless of diabetes status [216]. SGLT2i may be underprescribed in Blacks (and non-Blacks). Medicare FFS claims between 2012 and 2018 showed Black CKD patients with diabetes were less likely than non-Blacks (10.2 vs. 14.2%) to have started using SGLT2i and glucagon-like peptide 1 receptor agonists (GLP-1RA), another newer glucose-lowering medication with demonstrated cardiovascular and kidney protective benefits [217,218]. Analysis of the Optum Clinformatics Data Mart showed 17% lower odds of SGLT2i use in Black than White CKD patients [219]. Blacks were about half as likely to use SGLT2i (3.6% vs. 7.2% in Whites) but equally likely to use GLP-1RA as Whites (5.3 vs. 5.2%) among 2017-2020 NHANES participants with type 2 diabetes and eGFR≥30 [220]. The higher cost of these newer medications vis-a-vis more traditionally used therapies (e.g., $300 vs. $4/month for sulfonylurea) may be prohibitive in many Blacks and poorer Whites, leading to prescription abandonment [219]. This may explain why the odds for SGLT2i use were 2.17-fold greater under commercial insurance than Medicare Advantage [219]. However, Blacks were also less likely to be prescribed SGLT2i than Whites with CKD, diabetes, and atherosclerotic cardiovascular disease when treated by the Veterans Affairs Health Care system, for which high out-of-pocket cost would not be an issue (odds ratio of 0.87) [221]. An exception is the health equity initiatives of the Mass General Brigham, whose clinical departments are asked to track health disparities between Whites and minority groups [222]. A cross-sectional study of stages 3-5 adults in the Mass General Brigham CKD registry showed Blacks had 30% greater odds of being prescribed SGLT2i than Whites if they had diabetes, and 86% greater odds if they were non-diabetic [223].

Physician awareness

Nephrology referrals, and prescriptions of appropriate or contraindicated medications, depend upon the primary care physician’s awareness of underlying CKD. Documentation of international classification of disease (ICD) diagnostic codes provides a relatively good surrogate for primary care physician awareness [224]. Electronic records show Black patients with eGFR-defined CKD (two consecutive eGFRs between 15 and 60 mL/min/1.73m^2^ separated by at least 90 days) are more likely to receive an ICD CKD-diagnosis than White or non-Black patients in: a) 2011 Medicare fee-for-service claims for beneficiaries in ten eastern US states (1.93-fold greater odds) [225], b) a multi-site group practice of 11,774 patients with CKD stages 3 and 4 in eastern Massachusetts (2.71-fold greater odds) [226], and c) at all CKD stages in the Cleveland Clinic primary care physician (PCP) or nephrologist electronic data base (2.2-fold greater odds) [227]. Retrospective analysis of 270,170 patients in the Veterans Integrated Service Network 17 cohort showed diabetic and hypertensive Whites were significantly less likely to be recognized for CKD by an ICD code or nephrology referral than non-Whites [228]. Norton et al. [229] reported that 49.7% of Black Military Health System (MHS) beneficiaries aged 18 to 64 years with CKD who received care during fiscal years 2016 to 2018 had a diagnostic CKD code compared to 32.5% of Whites with CKD. The CDC reported that between 1999 and 2020, the proportion of CKD stages G3-G4 Whites who are personally aware of their condition has remained consistently lower than stages G3-G4 Blacks (e.g., 18.9% vs. 30.3%[230]).

Cystatin C 

Inexpensive screening for largely asymptomatic CKD is made possible by low cost of serum creatinine concentrations ($5.12). Over 250 million serum creatinine measurements are performed annually in the US [41]. GFR can also be estimated from serum concentrations of the low-molecular weight protein cystatin C (eGFR_cys_). Cystatin C has the advantages of being produced by all nucleated cells, not just skeletal muscle [231]. eGFR_cys_ is highly correlated with 2009 CKD-EPI eGFR_cr_, has accuracy similar to that of 2009 CKD-EPI eGFR_cr_, and like 2009 CKD-EPI, eGFR_cys_ is strongly associated with adverse clinical outcomes [232,233]. eGFR estimated from creatinine only and from cystatin C only: 1) produce nearly indistinguishable Kaplan-Meier estimates of the time to achieve an eGFR<20 mL/min/1.73m^2^ [234] and 2) produce very similar hazard ratios for KFRT [42], CKD progression [235] and all-cause mortality [235]. eGFR_cys_ has been advocated for being less dependent on race; however, this would currently cost an additional $18.75/sample [41]. A better GFR estimate includes both creatinine and cystatin in its estimation, but this includes a highly significant (p<10^-16^) race correction factor of 8% if Black [16] that is deftly ignored. Even a 50% cost-reduction in cystatin C measurement would still add an additional 2.3 billion dollars annually to the cost of CKD screening in order to obtain slightly poorer GFR estimate that gives the same expected result as 2009 CKD-EPI eGFR for both Blacks and non-Blacks (both estimating the same mGFR [16]) without offending racial sensitivities.

A better alternative

In casting aside the pioneering work by Levey and colleagues [4,7], the Task Force has sought to mandate a revised eGFR (2021 CKD-EPI) that discriminates against the CKD patient majority, antiquates drug dosing, thwarts trend-analyses of patient histories, complicates epidemiological interpretations of population temporal trends, provides no improvement of mGFR estimation [25], causes confusion when eGFR documentation is lacking [204], attenuates racial differences in eGFR-estimated risks for kidney failure and all cause and cardiovascular mortality [198]; incentives litigation for alleged past discrimination [236], expands Black while reducing non-Black prisoner compassionate release [237], and has been rejected by European community [238]. Inaccurate classification of CKD can lead to harm, i.e., overestimating mGFR can lead to inadequate CKD treatment whilst underestimating mGFR may increase costs or adverse events from to CKD medications and denied use of contrast agents, metformin, cancer treatments, and other medications [71,239]. The best eGFR for both Blacks and non-Blacks is the one that most accurately estimates mGFR. 

**Figure 6:**
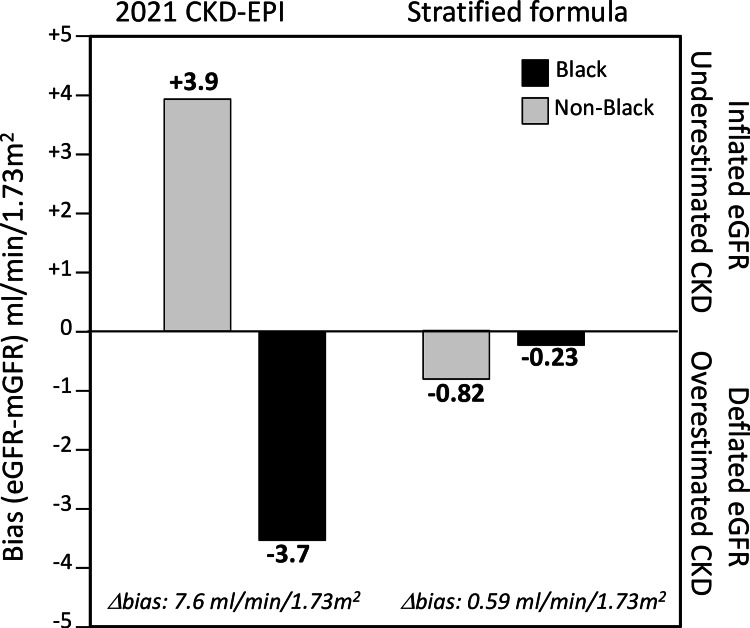
Race-specific bias (eGFR–mGFR) for the race-free 2021 CKD-EPI equation vs. deriving separate equations for Black and non-Black patients Adapted from the 100% and 0% Black analyses in Figure S9 of [16]

Statistically and scientifically, the best linear unbiased estimates for eGFR would be obtained from separate equations for Blacks and non-Blacks using age, sex and serum creatinine concentrations to predict mGFR, and to derive these estimates separately from Black and non-Black samples (Figure 6). Using a race-stratified algorithm to estimate mGFR instead of a race-adjusted algorithm yields the most accurate and essentially unbiased estimated for both Blacks and non-Blacks (Figure 5S in reference [16]), ensures that coefficients are derived exclusively from the relevant populations, and avoids the appearance of discrimination. Specifically, the 2021 validation dataset shows that eGFR_cr_ derived from stratified data would be negligibly biased in both Blacks and non-Blacks (estimated as -0.23 and -0.82 mL/min/1.73m^2^, respectively) with high accuracy (P_30_ of 87.2 and 88.9, respectively, corresponding to the 100% and 0% Black analyses in Figure S9 of reference [16]).

Stratification would also accommodate the smaller race difference in females than males [6,238,240], and eliminate the effect of the Black and non-Black sample proportions on the regression coefficients. Such an approach has been advocated for prescribing warfarin dosage [241] and treating heart failure hydralazine and isosorbide dinitrate [242] in European and African Americans. In addition to providing the minimum biased estimate for both Black and non-Black patients, stratification would preserve the physicians’ duty to provide the best medical treatment to all patients without withholding appropriate care to non-Blacks.

## Conclusions

The most common arguments cited for the abolition of the race correction factor were that race is a sociological rather than biological construct (statistically irrelevant), that higher average muscle mass in Blacks than Whites is unsubstantiated (much discussed, but also irrelevant), and that eliminating race corrections qualifies approximately one-million more Blacks for treatment (ignoring its impact on ten million non-Blacks who may be disqualified for treatments). Retaining the race correction factor does not negate the fact that many Blacks lack insurance and medical care which limit their access to diagnosis and treatment for a chronic health issue, and have higher rates of high blood pressure, diabetes, obesity, and heart disease, all of which increase CKD risk. Nor does its retention moderate the importance of patient medical history, BMI, dietary intake, genetic history, and existing chronic conditions when interpreting GFR diagnosis and CKD treatment.

The NKF/ASN Task Force cited “race as a social construct” and the “national call for re-evaluation of the use of race in clinical algorithms” as reasons for removing the race correction factor. There is a duty to deliver the best health care to all patients, and to base treatment decisions on the best science available. To that end, the current review has sought to highlight what was not wrong about the 2009 CKD-EPI eGFR_cr_ formula and to identify what is not right about the 2021 CKD-EPI eGFR_cr_ formula. It suggests a strong case can be made for retaining the race-corrected 2009 CKD-EPI formula. Setting politics aside, Figure 6 suggests that the unreleased race-specific formula appears to provide the best (i.e., least biased) estimate of mGFR for both Black and non-Black patients.

## Appendices

Equations for estimating measured glomeruler filtration rate (eGFR) from serum creatinine concentrations (Scr).

*Equation 1*. Modification of Diet in Renal Disease formula (MDRD, Eq 4) [4]

eGFR = 175 x Scr^-1.154^ x age^-0.203^ x 1.212 [if black] x 0.742 [if female]

*Equation 2*. 2009 Chronic Kidney Disease Epidemiology Collaboration (2009 CKD-EPI) [7]

eGFR = 141 x min(Scr/κ, 1)^α^ x max(Scr/κ, 1)^-1.209^ x 0.993Age x 1.018 [if female] x 1.159 [if black],

where Scr is serum creatinine, κ is 0.7 for females and 0.9 for males, α is -0.329 for females and -0.411 for males, min indicates the minimum of Scr/ κ or 1, and max indicates the maximum of Scr/κ or 1. 

*Equation 3*. 2021 Chronic Kidney Disease Epidemiology Collaboration retrofit (2021 CKD-EPI) [16]

eGFR = 142 x min(Scr/k,1)^α^ x max(Scr/k,1)^-1.200^ x 0.9938age x 1.012 [if female] 

where Scr is serum creatinine, k is 0.7 for females and 0.9 males, α is -0.241 for females and -0.302 for males, min indicates the minimum of Scr/k or 1, max indicates the maximum of Scr/k or 1
